# Genome-wide association mapping of soybean chlorophyll traits based on canopy spectral reflectance and leaf extracts

**DOI:** 10.1186/s12870-016-0861-x

**Published:** 2016-08-04

**Authors:** Arun Prabhu Dhanapal, Jeffery D. Ray, Shardendu K. Singh, Valerio Hoyos-Villegas, James R. Smith, Larry C. Purcell, Felix B. Fritschi

**Affiliations:** 1 Division of Plant Sciences, University of Missouri, Columbia, MO 65211 USA; 2 Crop Genetics Research Unit, USDA-ARS, 141 Experiment Station Road, Stoneville, MS 38776 USA; 3 Crop Systems and Global Change Lab, USDA-ARS, Beltsville, MD 20705 USA; 4 Forage Improvement Group, Lincoln Science Centre, Christchurch, 8140 New Zealand; 5 Department of Crop, Soil, and Environmental Sciences, University of Arkansas, Fayetteville, AR 72704 USA

**Keywords:** Abiotic stress tolerance, Chlorophyll *a*, Chlorophyll *b*, Chlorophyll *a/b* ratio, Total chlorophyll, Genome-wide association mapping, Single nucleotide polymorphisms, High-throughput phenotyping

## Abstract

**Background:**

Chlorophyll is a major component of chloroplasts and a better understanding of the genetic basis of chlorophyll in soybean [*Glycine max* (L.) Merr.] might contribute to improving photosynthetic capacity and yield in regions with adverse environmental conditions. A collection of 332 diverse soybean genotypes were grown in 2 years (2009 and 2010) and chlorophyll *a* (eChl_A), chlorophyll *b* (eChl_B), and total chlorophyll (eChl_T) content as well as chlorophyll a/b ratio (eChl_R) in leaf tissues were determined by extraction and spectrometric determination. Total chlorophyll was also derived from canopy spectral reflectance measurements using a model of wavelet transformed spectra (tChl_T) as well as with a spectral reflectance index (iChl_T).

**Results:**

A genome-wide associating mapping approach was employed using 31,253 single nucleotide polymorphisms (SNPs) to identify loci associated with the extract based eChl_A, eChl_B, eChl_R and eChl_T measurements and the two canopy spectral reflectance-based methods (tChl_T and iChl_T). A total of 23 (14 loci), 15 (7 loci) and 14 SNPs (10 loci) showed significant association with eChl_A, eChl_B and eChl_R respectively. A total of 52 unique SNPs were significantly associated with total chlorophyll content based on at least one of the three approaches (eChl_T, tChl_T and iChl_T) and likely tagged 27 putative loci for total chlorophyll content, four of which were indicated by all three approaches.

**Conclusions:**

Results presented here show that markers for chlorophyll traits can be identified in soybean using both extract-based and canopy spectral reflectance-based phenotypes, and confirm that high-throughput phenotyping-amenable canopy spectral reflectance measurements can be used for association mapping.

**Electronic supplementary material:**

The online version of this article (doi:10.1186/s12870-016-0861-x) contains supplementary material, which is available to authorized users.

## Background

Soybean (*Glycine max* [L.] Merr.) is the world’s most widely grown legume crop and produces high quality grain which contains 35–55 % easily digestible protein, 17–27 % oil, and about 30 % carbohydrates, among others components [1, 2]. Photosynthesis during the reproductive stages is positively correlated with crop yield, and improving the photosynthetic capacity of leaves has been suggested as a way to increase crop yields [3, 4]. Solar radiation is absorbed by the antenna pigments in chloroplasts and the excitation energy is directed to the reaction center pigments through resonance energy transfer to drive photochemical processes [5]. Chlorophylls *a* and *b* (Chl *a*, Chl *b*) represent the majority of the antenna complex pigments and thus are of great importance for light absorption, oxygen evolution, and conversion of light energy to chemical energy. In fact, the amount of solar radiation that is absorbed by a leaf is closely related to its chlorophyll concentration [6–8], which generally is positively related with photosynthetic rate [9–11]. Although not at all developmental stages, positive correlations between leaf chlorophyll concentration and photosynthesis have been reported for soybean [11–13], including correlation coefficients as high as ~0.7–0.9 during R4 and R5 developmental stages [11].

Leaf pigments are commonly quantified using extract-based methods [14–16] but can also be assessed with non-destructive techniques. Quantification by extract-based methods often involves the collection of leaf disks, solvent-based pigment extraction, and analysis by spectrophotometry or liquid chromatography. Alternatively, spectral reflectance based methods may be used to assess pigment composition and content of intact leaves and/or canopies [17, 18]. In fact, investigations into the relationships between plant characteristics and spectral reflectance have produced numerous models and spectral indices to predict a range of plant phenotypes, including chlorophyll content [17, 18–22]. Spectral reflectance measurements can be made in controlled environments as well as in field conditions, are quick, and can be repeated on the same sampling area to assess temporal dynamics. In addition, in contrast to extract-based methods, spectral reflectance characteristics of plant tissue can be assessed across a broad range of spatial scales from sub-leaf to plant and field levels. As such, spectral reflectance based methods have attracted much attention for high-throughput plant phenotyping [14, 23–25]. Given their role in light absorption, leaf and/or canopy spectral reflectance based methods are particularly promising for the assessment of chlorophylls [14, 16, 26].

Even though previous reports [27–29] indicate a considerable amount of genetic variation for chlorophyll characteristics in the soybean germplasm, only limited information on the genetics of soybean chlorophyll characteristics is available to date. Much of this information is based on mutants with chlorophyll-deficient phenotypes, several of which have been mapped [30–32]. In addition, Li et al. [33] mapped a total of 20 quantitative trait loci (QTL) for chlorophyll content determined using a chlorophyll meter (SPAD meter) at different developmental stages based on data collected from one location in 1 year and two locations in a second year. However, only one common QTL each was found in the same year across the two locations and across the 2 years in one location. More recently, Hao et al. [34] conducted genome-wide association analyses of chlorophyll and chlorophyll fluorescence parameters on a population of 168 soybean genotypes and identified 28 SNPs associated with chlorophyll content determined using a SPAD meter. Interestingly, for this study, Hao et al. determined the phenotypes when plants were at the full seed developmental stage (R6), by which time leaf traits including photosynthesis and chlorophyll levels are generally considerably reduced [35, 36]. Since leaf chlorophyll concentrations can change substantially over the course of plant and leaf development and are influenced by environmental and management factors, genotype by environment interactions, as observed by Hao et al. [34] and suggested by the results of Li et al. [33], are expected.

The vast majority of plant physiological traits are quantitative in nature [37]. Quantitative trait analysis can be used to unravel the interactions of complex traits for plant physiologists and breeders [38]. Probably because gas exchange measurements are very laborious and phenotypes are greatly influenced by environments during growth and measurement, the number of QTL studies for photosynthetic traits are relatively limited [39–41]. Nonetheless, genetic determinants of photosynthesis have been estimated in several species including wheat (*Triticum aestivum*) [42], maize (*Zea mays*) [43] and pea (*Pisum sativum*) [44]. In both wheat and pea, photosynthetic activity is controlled by additive gene action [42, 44]. In pea, the chlorophyll content is also governed by a preponderance of additive effects. For soybean, Li et al. [33] observed additive gene effects for chlorophyll content in F_2:3_ and F_2:4_ populations, and there is evidence that soybean breeding improved leaf-level photosynthetic rates in Canadian and Chinese cultivars [45, 46]. In contrast, Koester et al. [47] did not find a consistent increase in maximum photosynthetic capacity for US cultivars released between 1923 and 2007; however, light interception, radiation use efficiency, and harvest index did increase with year of cultivar release [48]. Interestingly, chlorophyll content of sunlit, fully expanded leaves at R5 increased with year of release for these cultivars. Hence, a better understanding of the genetic complexity of chlorophyll dynamics in soybean and application of molecular markers to identify QTLs associated with photosynthesis and photosynthesis-related traits may allow for continued improvement in photosynthesis and yields.

To date, no genome-wide association mapping study of total chlorophyll content based on high-throughput-amenable canopy spectral reflectance measurements has been published. In addition, a direct comparison of genetic loci identified for extract-based and spectral reflectance based chlorophyll traits is absent in the literature. Therefore, the objective of this research was to use genome-wide association mapping to identify genomic loci associated with i) extract-based measurements of chlorophyll *a*, chlorophyll *b*, and total chlorophyll content, as well as chlorophyll *a/b* ratio, and ii) two canopy spectral reflectance-based indices for total chlorophyll content in soybean.

## Methods

### Experimental design

No specific permission was required for the field study as it was conducted at the University of Missouri Bradford Research Center.

Field experiments were conducted in 2009 and 2010 at the Bradford Research Center (BRC) in Columbia, MO USA (38° 53′N, 92° 12′ W). A total of 385 maturity group IV soybean genotypes were grown on a Mexico silt loam soil (fine, montmorillonitic, thermic Typic Albaqualfs) in a randomized complete block design with three replications. Soybean were planted at a density of 25 seeds m^-2^ on 23 May 2009 and 27 May 2010 in four-row plots measuring 4.87 m in length and 3.04 m in width. The crop was managed according to standard agronomic practices as previously described [17]. The genotypes included in this study consisted of plant introductions that were selected from the USDA Germplasm Collection according to criteria in Dhanapal et al. [49, 50]. Genome-wide association analyses for chlorophyll traits were conducted on 332 of the 385 genotypes grown.

### Chlorophyll content determinations

Chlorophyll contents were determined using extract- and canopy reflectance-based methods. The chlorophyll contents determined from extracts of leaf disks are hereafter referred to as chlorophyll *a* (eChl_A), chlorophyll *b* (eChl_B), chlorophyll *a/b* ratio (eChl_R) and total chlorophyll (eChl_T). The two total chlorophyll contents derived from canopy spectral reflectance are hereafter referred to as i) spectral reflectance index total chlorophyll content (iChl_T), and ii) wavelet transformed spectral reflectance total chlorophyll content (tChl_T). A list of these traits along with their acronyms is provided in Table 1. Briefly, at 54 days after planting (DAP; 2009) and 60 DAP (2010), five 0.68 cm^2^ leaf disks were collected from the upper-most fully expanded, sun-exposed leaf (3rd or 4th leaf from the stem apex) from five different plants at flowering [R1-R2 stage, [51]]. The leaf disks were immediately placed in opaque glass vials containing 5 mL of ethanol (95 %, v/v). Samples were incubated at room temperature in the dark for 24 h, after which, the vials were vigorously agitated. A 200 μL aliquot of each sample was transferred to a 96 well-plate (Costech Analytical Technologies Inc., CA USA) and absorbance measured at 664, 648, and 470 nm on a Scanning Monochromatic Spectrophotometer (Bio-Tek PowerWave X 340 Microplate Reader, BioTek U.S. VT, USA). Total chlorophyll (eChl_T), chlorophyll *a* (eChl_A), and chlorophyll *b* (eChl_B) were calculated according to Lichtenthaler [52], expressed on a leaf-area basis (μg cm^-2^). The ratio of eChl_A and eChl_B was determined and is referred to as eChl_R.

**Table 1:** List of traits used in this study along with their acronyms

Data analysis	Acronym	References
Extraction and spectrophotometric measurements		
Chlorophyll a	eChl_A	Lichtenthaler 1987
Chlorophyll b	eChl_B	Lichtenthaler 1987
Total Chlorophyll	eChl_T	Lichtenthaler 1987
Chlorophyll a/b ratio	eChl_R	Lichtenthaler 1987
Canopy spectral reflectance based measurements		
Wavelet transformed spectral reflectance of total chlorophyll	tChl_T	Singh et al. 2013
Literature-based canopy spectral reflectance measurements		
Spectral total chlorophyll index	iChl_T	Gitelson et al. 2005

To match extract-based chlorophyll content determinations with chlorophyll assessments based on canopy spectral reflectance characteristics, reflectance measurements were conducted between 54 and 57 DAP in 2009 and 58 and 61 DAP in 2010 as described by Singh et al. [17]. In brief, for each plot, three random spectral reflectance measurements were collected using an ASD FieldSpec, FR spectroradiometer (Analytical Spectral Devices Inc., Boulder, CO, USA). The fiber optic cable was positioned about 0.5 m above the plant canopy and three reflectance spectra (350 to 1800 nm) were collected and averaged for each plot.

Chlorophyll contents were calculated based on reflectance spectra from i) the ratio of the area under the curve in the 840–870 nm region and the 720–730 nm region [∫R_840-870_/∫R_720-730_] [53] for iChl_T, and ii) a model developed by Singh et al. [17] for tChl_T. Singh et al. [17] used the extract-based total chlorophyll content (eChl_T) data to test multiple models for total chlorophyll estimation based on canopy spectral reflectance measurements [17]. Among the tested models [17], one based on multiple linear regression (MLR) analysis and incorporating six wavebands derived from continuous wavelet transformed spectral reflectance data using the ‘Mexican hat’ wavelet family, most accurately predicted eChl_T. Consequently, this model was used to estimate tChl_T.

### Descriptive statistics and BLUP calculation

All descriptive statistics and Pearson correlation analyses were conducted for each variable (eChl_A, eChl_B, eChl_R, eChl_T, tChl_T and iChl_T) using PROC MEAN and PROC CORR procedures of SAS Version 9.3 (SAS Institute Inc., Cary, NC, USA). Variance components were determined using the PROC MIXED of SAS [54, 55] as described in Dhanapal et al. [49], considering all effects as random. Broad sense heritability estimates for all variables were derived using variance components obtained from the PROC MIXED procedure of SAS Version 9.3 as previously reported [50, 56, 57]. Best linear unbiased prediction (BLUP) values were used to reduce error variance. For each variable, data from both years were used to calculate one BLUP value to represent each genotype for GWAS analysis.

### Kinship matrix and population structure

The genome-wide association mapping software TASSEL 5.2.3 was used to create a kinship matrix (K). All 31,253 polymorphic SNPs were used for generation of K based on the scaled Identity by State (IBS) similarity method as described [58]. The software program STRUCTURE 2.2 [59] was used to infer the population structure based on ten independent iterations with 1 to 10 hypothetical sub-populations with an admixture and allele frequency correlated model. The correct estimation of *k* (*k* = 8) was provided by joining the log probability of data [LnP(D)] from the STRUCTURE output and an *ad hoc* statistic Δ*k*, determined by the value at which LnP(D) reached a plateau as described in [60].

### SNP genotyping and genome wide association mapping

Genotypic data from the SoySNP50K iSelect SNP Beadchip [61] are publicly available at Soybase (http://www.soybase.org/snps/download.php) and were obtained for the 332 soybean accessions and used in this study. For genome-wide association mapping of eChl_A, eChl_B, eChl_R, eChl_T, tChl_T and iChl_T, 31,253 polymorphic SNPs with a minor allele frequency (MAF) ≥ 5 % across the 332 genotypes were used.

Genome-wide association mapping was conducted based on the BLUP values using a mixed linear model with Q-matrix and K-matrix (MLM + Q + K). The Q and K matrices were used as corrections for population structure and/or genetic relatedness to help avoid false positives [50, 62, 63].

Genome-wide association mapping based on the MLM + Q + K model was conducted with TASSEL 5 [64, 65]. Multiple testing was performed using QVALUE R 3.1.0, employing the smoother method [66], an extension of the false discovery rate (FDR) method [67], to assess the significance of marker-trait associations. All markers that satisfied multiple testing had –log10 *P* values ≥ 3.2, which is above the threshold used by others for soybean [68–70]. Markers with FDR < 0.05 were considered significant [71, 72].

## Results

### Environmental conditions, chlorophyll phenotypes, and broad-sense heritability

In general, environmental conditions for the period from planting through collection of leaf disks and canopy spectral reflectance measurements were similar between the 2 years and close to 30-year averages. Daily average temperatures between planting and leaf-disk sampling were somewhat higher in 2010 (24.73 °C) than in 2009 (22.88 °C). The observed differences in temperatures were mirrored by higher solar radiation in 2010 (21.77 MJ m^-2^) than in 2009 (20.50 MJ m^-2^). Precipitation totals for the months encompassing planting through collection of leaf disks and canopy spectral reflectance measurements (May, June, and July) were the same or greater in 2009 and 2010 than the 30 year averages. Cumulative precipitation for May, June, and July was 418, 494, and 319 mm for 2009, 2010, and the 30-year average, respectively, and irrigation was therefore not necessary to avoid drought stress in either year.

**Fig. 1:**
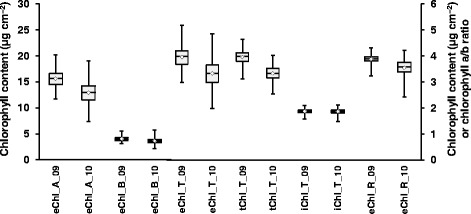
Box plot showing differences in chlorophyll *a* (eChl_A), total chlorophyll (eChl_T) and chlorophyll *b* (eChl_B) using the extract-based chlorophyll method and total chlorophyll using extractable chlorophyll method (eChl_T), wavelet transformed spectral reflectance chlorophyll method (tChl_T) and spectral reflectance index total chlorophyll method (iChl_T). Box edges represent the upper and lower quantile with median value shown as bold line in the middle of the box and mean values as white diamonds. Left scale represent the values for eChl_A, eChl_T and tChl_T. Right scale represents the values for eChl_R, eChl_B and iChl_T

The 332 MG IV soybean genotypes varied widely for the different chlorophyll traits (Fig. 1). Analysis of variance indicated significant year effects for all six traits (*P* < 0.0001). However, except for the chlorophyll *a/b* ratio (eChl_R), no genotype by year interactions were observed. For all traits, mean and median values were larger in 2009 than 2010. The ranges in chlorophyll contents in 2009 were smaller than in 2010 for all traits except tChl_T. Across the 2 years, the range in chlorophyll content was largest for eChl_T (9.85 μg cm^-2^) followed by eChl_A (7.91 μg cm^-2^), tChl_T (6.06 μg cm^-2^), eChl_B (2.39 μg cm^-2^), and a considerably smaller range for iChl_T (0.51 μg cm^-2^). The eChl_R, the only trait for which a genotype by year interaction was observed, ranged from 2.94 to 4.26 (μg cm^-2^) across the 2 years. Correlation coefficients for each trait between the 2 years ranged from 0.35 for eChl_A to 0.45 for tChl_T and were highly significant (*P* ≤ 0.001), except for eChl_R which was 0.11 but nonetheless significant (*P* < 0.05).

The relationships among all chlorophyll traits, including extract-based and canopy spectral reflectance based determinations, were examined by correlation analysis based on across-year genotypic averages. As expected, strong positive correlations were observed between eChl_A and eChl_B (*r* = 0.90), eChl_A and eChl_T (*r* = 0.95), eChl_B and eChl_T (*r* = 0.94). The two canopy-based reflectance methods for total chlorophyll content were positively correlated with extract-based total chlorophyll content (tChl_T and eChl_T, *r* = 0.67; iChl_T and eChl_T, *r* = 0.48) and also showed significant positive correlations with extract-based chlorophyll *a* and chlorophyll *b* measurements (Table 2). However, as illustrated by the big difference in iChl_T compared to eChl_T based chlorophyll contents, the index applied to the canopy spectral reflectance measurements to calculate iChl_T, did not predict well the absolute values of eChl_T. Calculations of broad-sense heritability indicated the highest heritability for tChl_T (62 %) followed by iChl_T (59 %), eChl_B (56 %), eChl_T (49 %), eChl_A (46 %) and eChl_R (15 %).

**Table 2:** Pearson correlation coefficients for extractable chlorophyll traits chlorophyll *a* (eChl_A), chlorophyll *b* (eChl_B), total chlorophyll (eChl_T) and chlorophyll *a/b* ratio (eChl_R) and wavelet transformed spectral reflectance total chlorophyll (tChl_T) and spectral reflectance index total chlorophyll (iChl_T)

	eChl_A	eChl_B	eChl_R	eChl_T	tChl_T	iChl_T
eChl_A		0.90^***^	0.41^***^	0.95^**^	0.65^***^	0.47^***^
eChl_B			−0.01^ns^	0.94^***^	0.69^***^	0.49^***^
eChl_R				0.33^***^	0.03 ^ns^	0.01 ^ns^
eChl_T					0.67^***^	0.48^***^
tChl_T						0.70^***^

The symbols ***, **, * and ns represent the significance level of *P* ≤ 0.001, *P* ≤ 0.01, *P* ≤ 0.05 and not significant (*P* > 0.05)

### Genome-wide association mapping

With the exception of eChl_R no significant genotype by year interactions were observed. Therefore, BLUP values across years were calculated for each chlorophyll trait and used for genome-wide association mapping. Analysis was conducted with 31,253 SNP markers and the extractable chlorophyll traits including eChl_A, eChl_B and eChl_R and eChl_T and two canopy-based reflectance methods for total chlorophyll (tChl_T and iChl_T) was conducted using an MLM + Q + K model using TASSEL 5.2.3 software. The K (kinship matrix) and Q (population structure) were used as corrections for genetic relatedness and population structure to help avoid false positives [63, 73]. Application of *q*FDR < 0.05 reduced the number of SNPs from 31,253 to 23, 15, 26 and 14 unique candidate SNPs associated with 14, 7, 15 and 10 putative genomic loci for eChl_A, eChl_B, eChl_T and eChl_R, respectively, and 20 and 18 unique candidate SNPs showed association with 12 and 11 putative loci for tChl_T and iChl_T, respectively (Additional file 1: Table S1 and Additional file 2: Table S2).

**Fig. 2:**
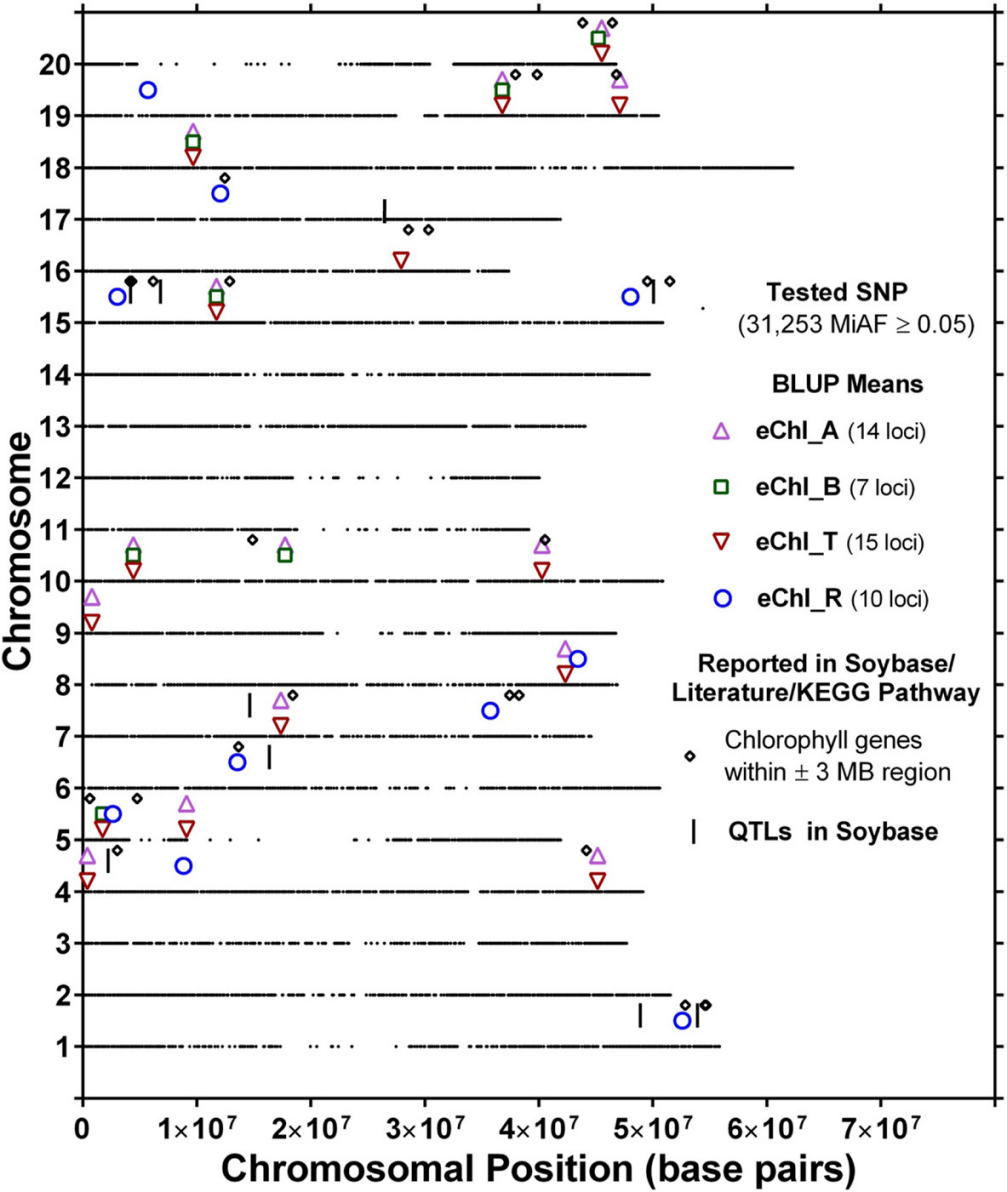
Location of putative loci significantly associated with extractable chlorophyll *a* (eChl_A), chlorophyll *b* (eChl_B), total chlorophyll (eChl_T) and chlorophyll *a/b* ratio (eChl_R) and 28 chlorophyll-related genes identified in Soybase, literature search and KEGG pathway of chlorophyll biosynthesis. Loci are indicated by upward triangles, squares, downward triangles and circles positioned above the respective chromosome. For each chromosome, the black dots represent the locations of SNPs evaluated for association with eChl_A, eChl_B, eChl_R and eChl_T

Association analysis for eChl_A identified a total of 23 significant SNPs. Since SNPs in close proximity probably identify the same locus, these 23 unique SNPs likely mark 14 putative loci (Fig. 2). The R^2^ for these loci ranged from 3.7 to 6.1 % (Additional file 1: Table S1). The putative eChl_A locus on chromosome 18 was identified by seven closely spaced SNPs and the one on chromosome 20 by three SNPs. One of two loci on chromosome 19 was identified by two SNPs while the remaining eleven loci were marked by one SNP each.

Fifteen unique SNPs were identified as having significant associations with eChl_B. Based on their genomic position, these 15 SNPs likely identified seven putative loci with R^2^ ranging from 3.1 to 6.1 % (Fig. 2) (Additional file 1: Table S1). The putative eChl_B locus on chromosome 18 was identified by five closely spaced SNPs, one locus on chromosome 15 was identified by four SNPs, and the remaining six loci were identified by a single SNP significantly associated with eChl_B.

For eChl_R, association analysis indicated 14 significant SNPs. Together these 14 SNPs likely identified 10 putative loci with R^2^ ranging from 3.6 to 6.3 % (Additional file 1: Table S1). Six of these loci were identified by a single SNP each (Fig. 2). Putative loci located on chromosomes 1, 4, and 19, and one of the two loci on chromosome 15, were identified by two closely spaced SNPs.

**Fig. 3:**
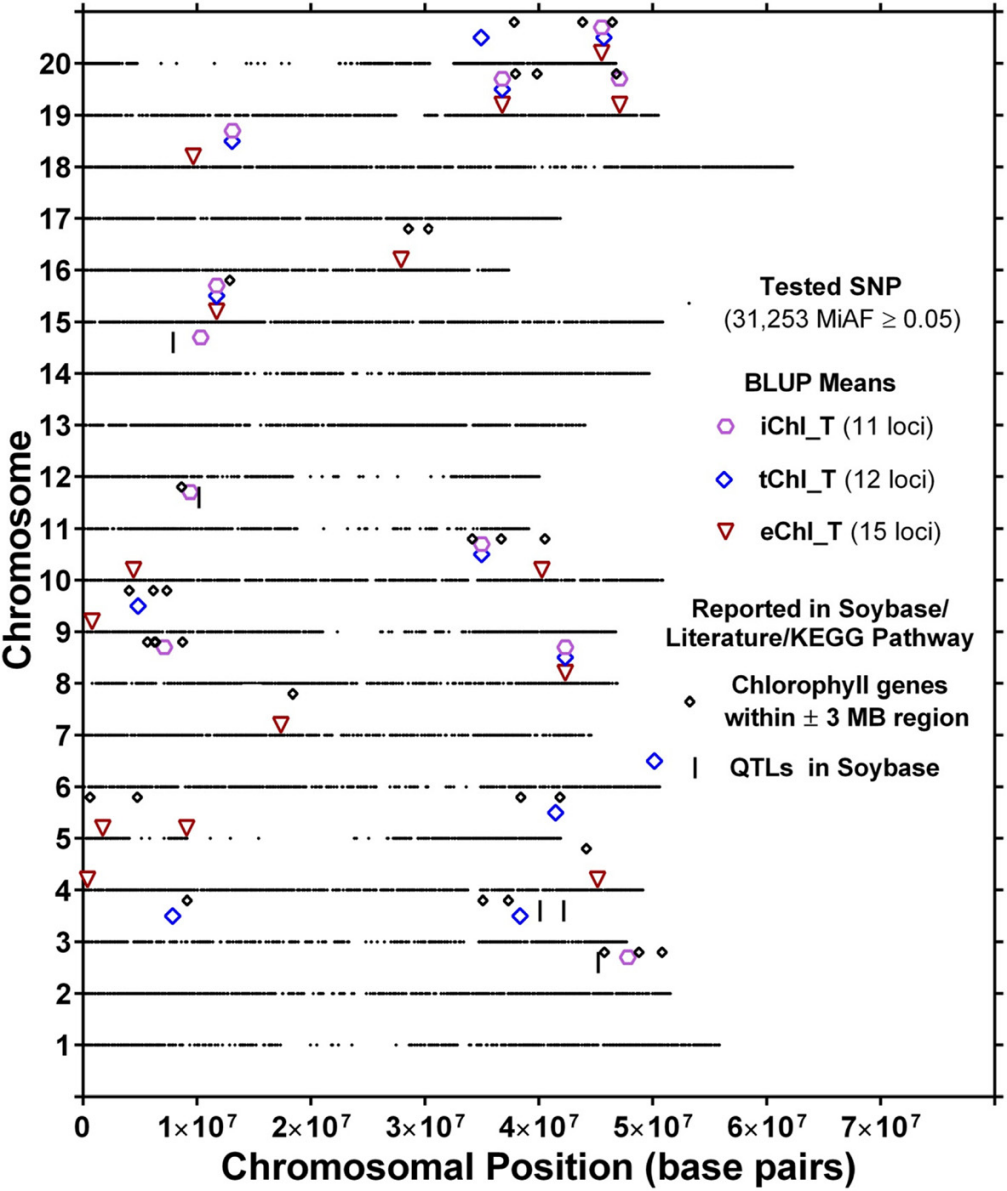
Location of putative loci significantly associated with total chlorophyll (eChl_T, tChl_T and iChl_T) and 32 chlorophyll related genes identified in Soybase, literature search and KEGG pathway of chlorophyll biosynthesis. Loci are indicated by hexagrams, large diamonds and downward triangles positioned above the respective chromosomes. For each chromosome, the black dots represent the locations of SNPs evaluated for association with total chlorophyll (eChl_T, tChl_T and iChl_T)

A total of 26 unique SNPs were significantly associated with eChl_T phenotypic BLUP values, identifying a total of 15 putative loci (Fig. 3). The R^2^ for these putative loci ranged from 3.4 to 6.1 % (Additional file 2: Table S2). One putative locus on chromosome 18 was identified by seven closely spaced SNPs and, one on chromosome 20 was identified by four closely spaced SNPs, while one of two loci each on chromosome 19 and 15 were identified by two closely spaced SNPs. The remaining eleven loci were identified by one SNP each, showing significant association for eChl_T.

Genome-wide association analysis for the two canopy spectral reflectance based methods used for total chlorophyll determination resulted in the identification of 20 (tChl_T) and 18 (iChl_T) candidate SNPs, representing 12 and 11 putative loci, respectively (Fig. 3). The R^2^ for the putative loci ranged from 3.6 to 6.0 % for tChl_T and from 3.3 to 6.0 % for iChl_T (Additional file 2: Table S2). The 20 SNPs significantly associated with tChl_T marked 12 putative loci of which one, located on chromosome 20, was identified by five closely spaced SNPs, and one locus on chromosome 5 was identified by three closely spaced SNPs. One locus each on chromosome 8 and 18 were identified by two SNPs, and the remaining eight loci were identified by one SNP each showing significant association for tChl_T. The 18 unique SNPs significantly associated with iChl_T marked 11 putative loci of which seven were identified by single SNPs (Fig. 3) (Additional file 2: Table S2). One locus on chromosome 14 was identified by four closely spaced SNPs, one locus on chromosome 2 by three closely spaced SNPs, and one locus each on chromosome 18 and 20 by two closely spaced SNPs.

**Fig. 4:**
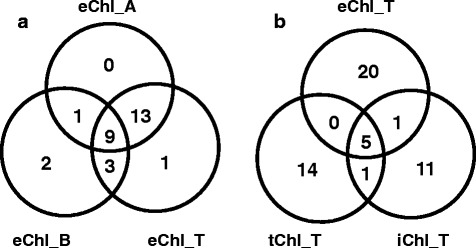
**a** Venn diagram showing the number of SNPs significantly associated with extractable chlorophyll *a* (eChl_A), chlorophyll *b* (eChl_B) and total chlorophyll (eChl_T). **b** Venn diagram showing the number of SNPs significantly associated with extractable total chlorophyll (eChl_T), wavelet transformed spectral reflectance total chlorophyll (tChl_T) and spectral reflectance index total chlorophyll (iChl_T)

Genome-wide association mapping for extract-based chlorophyll traits identified a total of 78 SNPs (23 + 15 + 14 + 26) with 43 unique putative candidate SNPs contributing to 14, 7, 10 and 15 putative loci for eChl_A and eChl_B, eChl_R and eChl_T, respectively (Additional file 1: Table S1 and Additional file 2: Table S2). The 78 SNPs marked 24 unique putative loci, seven of which were identified by three of the four extract-based chlorophyll traits. Eight of the 24 loci were identified by at least two of the four chlorophyll traits and the remaining nine loci were only identified by one of the four chlorophyll traits. None of the SNPs or loci identified for eChl_R overlapped with those found for eChl_A, eChl_B, or eChl_T. Examination of SNPs identified for eChl_A, eChl_B and eChl_T identified several that were detected based on two or three of these traits (Figs. 2 and 4a). Twenty-two SNPs were in common between eChl_A and eChl_T, 12 SNPs between eChl_B and eChl_T, 10 SNPs between eChl_A and eChl_B, and nine SNPs were common among all three traits (Fig. 4a) (Additional file 3: Table S3). One locus on chromosome 18 was identified by five closely spaced SNPs and one locus each on chromosomes 10, 19 and 20 was identified by one SNP, showing significant association with eChl_A, eChl_B and eChl_T.

Mapping of total chlorophyll content based on eChl_T, tChl_T, and iChl_T indicated a total of 64 significant SNPs (26 + 20 + 18). Of these 64 SNPs, five SNPs were identified based on all three methods, one SNP was in common between eChl_T and iChl_T only, and one SNP was in common between tChl_T and iChl_T only (Figs. 3 and 4b). Of the five total SNPs identified based on all three methods, one locus on chromosome 20 was identified by two closely spaced SNPs, and three loci, one each on chromosomes 15, 18 and 19, were identified by one SNP each that was in common for eChl_T, tChl_T and iChl_T (Fig. 3) (Additional file 2: Table S2 and Additional file 4: Table S4). Consequently, a total of 52 unique SNPs representing 27 putative loci were found. Four of the 27 putative loci were identified using all three total chlorophyll determination methods (one locus each on chromosomes 8, 15, 19 and 20). One putative locus each on chromosomes 10 and 18 was identified for tChl_T and iChl_T but not eChl_T. Another locus on chromosome 19 was identified for eChl_T and iChl_T but not tChl_T. The remaining 20 putative loci were all identified for only one of the three methods of total chlorophyll determination (Additional file 2: Table S2 and Additional file 4: Table S4).

### Identification of candidate SNPs and genes

All SNPs identified for eChl_A (23), eChl_B (15), eChl_R (14), eChl_T (26), tChl_T (20), and iChl_T (18) that satisfied the FDR < 0.05 were considered as the most promising candidate SNPs associated with chlorophyll contents or the Chl *a/b* ratio. Based on the 60 bp sequences flanking the 43 unique candidate SNPs for extract-based chlorophyll traits and 52 unique candidate SNPs for the three total chlorophyll content traits, a blast search was conducted with default parameters in Soybase (www.soybase.org) to identify putative candidate genes. The search for candidate genes found that, for extract-based chlorophyll traits, 12 SNPs were present in introns or coding regions of a gene, and that, for the three total chlorophyll content traits, 17 SNPs were present in introns, coding regions or 3’- untranslated regions (UTR) of a gene (Additional file 3: Table S3 and Additional file 4: Table S4). For all SNPs not located in a gene, the gene closest to the SNP was identified in Soybase and is listed in the supporting documents (Additional file 3: Table S3 and Additional file 4: Table S4). However, none of these genes have any obvious direct relationship with any of the chlorophyll traits. An additional search for candidate genes was performed in Soybase using the term “chlorophyll”, and soybean chlorophyll biosynthetic pathway (KEEG pathway http://www.genome.jp/kegg-bin/show_pathway?gmx00860). These searches revealed 155 chlorophyll-related genes from Soybase and 12 chlorophyll-related genes from the KEGG pathway (data not shown). Of these chlorophyll-related genes, 28 were located within ± 3 Mb [50, 69, 74, 75] of one of the 43 unique candidate SNPs identified for extract-based chlorophyll traits (Table 3), and 33 chlorophyll-related genes that were located within ± 3 MB of one of the 52 unique candidate SNPs identified for the three total chlorophyll content traits (Table 4).

**Table 3:** List of 28 known chlorophyll-related genes within a ± 3 MB region of the 43 putative candidate SNPs identified from Soybase (www.soybase.org) for extractable chlorophyll *a* (eChl_A), chlorophyll *b* (eChl_B), total chlorophyll (eChl_T) and chlorophyll *a/b* ratio (eChl_R)

Loci	Gene ^a^	Chromosome	Start	Stop	Soybase ^b^ /Pathway/Reference	Distance to SNP (Mb)	Functional annotation	Trait
1	Glyma01g41320	Gm01	528,55,359	528,56,591	Glyma 1.0	0.29	Chlorophyll A-B binding protein	eChl_R
	Glyma01g43630	Gm01	545,89,487	545,98,903	Glyma 1.0	1.80	Magnesium chelatase activity (chlorophyll biosynthetic process)	eChl_R
	Glyma01g43720	Gm01	546,67,132	546,69,898	Glyma 1.0	1.80	Tetrapyrrole biosynthetic process (Porphobilinogen deaminase)	eChl_R
	Glyma01g42390	Gm01	545,54,210	545,56,460	Fang et al 2014	1.80	Stay-Green (SGR) gene D2 (Chlorophyll catabolic process)	eChl_R
2	Glyma04g04110	Gm04	30,19,987	30,21,151	Glyma 1.0	2.64	Chlorophyll A-B binding protein	eChl_A
3	Glyma04g37740	Gm04	441,63,842	441,70,887	Glyma 1.1	0.98	regulation of transcription, DNA-templated	eChl_A and eChl_T
5	Glyma05g01000	Gm05	6,06,608	6,08,812	Glyma 1.0	1.11	Electron transfer flavoprotein-Ubiquinone oxidoreductase	eChl_B, eChl_R and eChl_T
6	Glyma05g05450	Gm05	47,64,696	47,66,688	Glyma 1.0	2.14	Chlorophyll A-B binding family protein	eChl_B, eChl_R and eChl_T
7	Glyma06g17360	Gm06	136,67,004	136,74,569	Glyma 1.0	0.13	Regulation of transcription, DNA-templated (ATP-dependent CLP protease)	eChl_R
8	Glyma07g18470	Gm07	184,08,168	184,13,639	Glyma 1.0	1.05	Prenyltransferase activity	eChl_A and eChl_T
9	Glyma07g32550	Gm07	374,27,501	374,30,163	KEGG pathway database	1.69	Magnesium chelatase activity (chlorophyll biosynthetic process)	eChl_R
	Glyma07g33320	Gm07	382,60,227	382,61,619	Glyma 1.0	2.52	UbiA prenyltransferase family (prenyltransferase activity)	eChl_R
12	Glyma10g13190	Gm10	148,88,257	149,01,045	Glyma 1.1	2.86	Pyridine nucleotide-disulphide oxidoreductase	eChl_B
13	Glyma10g32080	Gm10	405,24,508	405,27,468	Glyma 1.0	0.27	Chlorophyll A-B binding protein	eChl_A and eChl_T
15	Glyma15g05790	Gm15	41,14,634	41,16,181	Glyma 1.0	1.09	Chlorophyll A-B binding protein	eChl_R
	Glyma15g06050	Gm15	42,95,728	43,06,099	Glyma 1.0	1.28	Magnesium chelatase activity (chlorophyll biosynthetic process)	eChl_R
	Glyma15g08680	Gm15	61,55,823	61,58,347	Campbell et al 2015	2.93	Magnesium chelatase activity	eChl_R
16	Glyma15g16570	Gm15	128,69,848	128,76,153	Glyma 1.0	1.49	Magnesium chelatase activity (chlorophyll biosynthetic process)	eChl_A, eChl_B and eChl_T
17	Glyma15g42140	Gm15	495,29,893	495,34,600	Glyma 1.0	1.51	ATP-citrate synthase	eChl_R
	Glyma15g43150	Gm15	514,86,036	514,91,942	Reed et al 2014	2.96	Biogenesis of Photosystem I and II	eChl_R
18	Glyma16g24570	Gm16	285,47,662	285,50,487	Glyma 1.0	0.65	Chlorophyll catabolic process (Chlorophyllase.)	eChl_T
	Glyma16g26130	Gm16	303,09,204	303,11,593	Glyma 1.0	2.41	Chlorophyll A-B binding protein	eChl_T
19	Glyma17g15730	Gm17	124,56,729	124,58,671	Glyma 1.0	0.41	Chlorophyll A-B binding protein	eChl_R
21	Glyma19g30350	Gm19	379,57,536	379,60,664	Glyma 1.0	1.17	Oxidation-reduction process (Rubrerythrin)	eChl_A, eChl_B and eChl_T
22	Glyma19g32070	Gm19	398,43,036	398,49,603	Glyma 1.0	2.97	Magnesium chelatase activity (chlorophyll biosynthetic process)	eChl_A, eChl_B and eChl_T
23	Glyma19g40370	Gm19	467,94,372	467,99,578	Glyma 1.0	0.27	Magnesium chelatase activity (chlorophyll biosynthetic process)	eChl_A and eChl_T
24	Glyma20g35530	Gm20	438,24,060	43826992	Glyma 1.0	1.36	Chlorophyll A-B binding protein	eChl_A, eChl_B and eChl_T
	Glyma20g38941	Gm20	464,38,179	46439540	Glyma 1.1	0.92	Homogentisate phytyltransferase 1	eChl_A, eChl_B and eChl_T

^a^ As reported in Soybase

^b^ Annotation version information based on Soybase

**Table 4:** List of 33 known chlorophyll-related genes within a ± 3 MB region of the 52 putative candidate SNPs identified from Soybase (www.soybase.org) for three total chlorophyll determination methods namely extractable chlorophyll (eChl_T), wavelet transformed spectral reflectance chlorophyll (tChl_T) and spectral reflectance index total chlorophyll (iChl_T)

Loci	Gene ^a^	Chromosome	Start	Stop	Soybase ^b^/Pathway	Distance to SNP (Mb)	Functional annotation	Trait
1	Glyma02g40490	Gm02	45747413	457,63,801	Glyma 1.1	2.03	Mitochondrial Fe/S cluster exporter, ABC superfamily	iChl_T
	Glyma02g39990	Gm02	451,89,739	451,97,024	Glyma 1.1	2.61	Translocon at the inner envelope membrane of chloroplasts	iChl_T
	Glyma02g44150	Gm02	487,89,243	487,90,727	Glyma 1.1	0.99	Chlorophyll a biosynthetic process (geranylgeranyl reductase)	iChl_T
	Glyma02g47120	Gm02	508,18,647	508,20,935	Glyma 1.1	2.92	Red chlorophyll catabolite reductase (RCC reductase)	iChl_T
2	Glyma03g08280	Gm03	91,32,182	91,34,276	Glyma 1.1	1.28	Chlorophyll A-B binding protein	tChl_T
3	Glyma03g27380	Gm03	350,95,413	350,98,484	Glyma 1.1	2.96	Oxidation-reduction process (Rubrerythrin)	tChl_T
	Glyma03g29330	Gm03	373,17,236	373,23,794	Glyma 1.1	1.04	Magnesium chelatase activity	tChl_T
5	Glyma04g37740	Gm04	441,63,842	441,70,887	Glyma 1.1	0.98	Regulation of Transcription (ATP-dependent CLP protease)	eChl_T
6	Glyma05g01000	Gm05	6,06,608	6,08,812	Glyma 1.1	1.11	Electron transfer flavoprotein-ubiquinone oxidoreductase	eChl_T
7	Glyma05g05450	Gm05	47,64,696	47,66,688	Glyma 1.1	2.95	Chlorophyll A-B binding family protein	eChl_T
8	Glyma05g38510	Gm05	418,44,917	418,50,362	Glyma 1.1	0.40	Regulation of transcription, DNA-templated (ATP-dependent CLP protease)	tChl_T
	Glyma05g38570	Gm05	384,15,657	384,18,787	KEGG Pathway	2.98	Magnesium protoporphyrin IX methyltransferase activity (chlorophyll biosynthetic process)	tChl_T
10	Glyma07g18470	Gm07	184,08,168	184,13,639	Glyma 1.1	1.05	UbiA prenyltransferase family (prenyltransferase activity)	eChl_T
11	Glyma08g07880	Gm08	56,44,333	56,45,834	Glyma 1.1	1.49	Chlorophyll A-B binding protein	iChl_T
	Glyma08g08770	Gm08	62,68,835	62,70,396	Glyma 1.1	0.87	Chlorophyll A-B binding protein	iChl_T
	Glyma08g08920	Gm08	63,57,088	63,63,013	Glyma 1.1	0.78	Magnesium chelatase activity (chlorophyll biosynthetic process)	iChl_T
12	Glyma08g12070	Gm08	87,20,805	87,26,588	Glyma 1.1	1.59	Chlorophyllide a oxygenase [overall] activity	iChl_T
13	Glyma09g05240	Gm09	40,35,167	40,41,182	Glyma 1.1	0.80	Magnesium chelatase activity (chlorophyll biosynthetic process)	tChl_T
	Glyma09g07310	Gm09	61,57,184	61,57,688	Glyma 1.1	1.32	Chlorophyll A-B binding protein	tChl_T
14	Glyma09g08260	Gm09	73,39,820	73,42,548	Glyma 1.1	2.51	Chlorophyll A-B binding protein	tChl_T
15	Glyma10g25710	Gm10	341,46,057	341,58,388	KEGG Pathway	0.84	Coenzyme F420 hydrogenase	tChl_T and iChl_T
16	Glyma10g27890	Gm10	366,89,550	366,94,993	Glyma 1.1	0.84	Oxidation-reduction process (Protoporphyrinogen oxidase)	tChl_T and iChl_T
17	Glyma10g32080	Gm10	405,24,508	405,27,468	Glyma 1.1	0.27	Chlorophyll A-B binding protein	eChl_T
18	Glyma11g12110	Gm11	86,45,442	86,50,623	Glyma 1.1	0.75	Magnesium chelatase activity (chlorophyll biosynthetic process)	iChl_T
20	Glyma15g16570	Gm15	128,69,848	128,76,153	Glyma 1.1	1.49	Magnesium chelatase activity (chlorophyll biosynthetic process)	eChl_T and tChl_T
21	Glyma16g24570	Gm16	285,47,662	285,50,487	Glyma 1.1	0.65	Chlorophyll catabolic process (Chlorophyllase.)	eChl_T
	Glyma16g26130	Gm16	303,09,204	303,11,593	Glyma 1.1	2.41	Chlorophyll A-B binding protein	eChl_T
24	Glyma19g30350	Gm19	379,57,536	379,60,664	Glyma 1.1	1.17	Oxidation-reduction process (Rubrerythrin)	eChl_T, tChl_T and iChl_T
	Glyma19g32070	Gm19	398,43,036	398,49,603	Glyma 1.1	2.96	Magnesium chelatase activity (chlorophyll biosynthetic process)	eChl_T, tChl_T and iChl_T
25	Glyma19g40370	Gm19	467,94,372	467,99,578	Glyma 1.1	0.27	Magnesium chelatase activity (chlorophyll biosynthetic process)	eChl_T and iChl_T
26	Glyma20g28890	Gm20	378,38,174	378,39,638	Glyma 1.1	2.90	Chlorophyll A-B binding protein	tChl_T
27	Glyma20g35530	Gm20	438,24,060	438,26,992	Glyma 1.1	1.36	Chlorophyll A-B binding protein	eChl_T, tChl_T and iChl_T
	Glyma20g38941	Gm20	464,38,179	464,39,540	Glyma 1.1	0.73	Homogentisate phytyltransferase 1	eChl_T, tChl_T and iChl_T

^a^ As reported in Soybase

^b^ Annotation version information based on Soybase

## Discussion

### Chlorophyll phenotypes

Considerable variation in extract-based chlorophyll traits (eChl_A, eChl_B, eChl_T, and eChl_R) and canopy-based spectral reflectance total chlorophyll content traits (tChl_T and iChl_T) was observed among the 332 soybean genotypes (Fig. 1). The eChl_A, eChl_B, eChl_T, and eChl_R average values observed were similar to chlorophyll contents and chlorophyll *a/b* ratios reported previously for soybean [28, 76]. As expected, given that total chlorophyll is a function of chlorophyll *a* and chlorophyll *b*, the correlations of eChl_A and eChl_B with eChl_T were positive and very strong (Table 2). Positive relationships were also found among all three total chlorophyll traits, despite the fact that leaf disks extracted for eChl_T determination were collected from uppermost fully expanded, sun-exposed leaflets while the reflectance measurements used for tChl_T and iChl_T determination represented a canopy of leaves of different ages and positions on the plants. Both tChl_T and iChl_T were estimated based on the same canopy spectral reflectance measurements, but the two determinations were based on independent indices, one developed by Gitelson et al. [53], and the other by Singh et al. [17]. Nonetheless, the two canopy spectral reflectance based estimates were more closely related to each other than either of them was with eChl_T. Since tChl_T was estimated based on a model Singh et al. [17] developed using the eChl_T and canopy spectral reflectance measurements from the 332 genotypes examined in this study, the stronger positive correlation between eChl_T and tChl_T compared to eChl_T and iChl_T was expected (Table 2).

### Putative loci for extract-based chlorophyll traits and known chlorophyll genes in their vicinity

Advances in high-throughput genotyping technologies have enabled genome-wide association analysis to be a powerful tool for detection and mapping of quantitative trait loci (QTLs) underlying complex traits in soybean. The MLM + Q + K model applied in this study resulted in the identification of between 14 and 26 significant SNPs for each of the investigated chlorophyll traits. The majority of the SNPs identified for eChl_A, eChl_B, and eChl_T, were common between at least two of these traits, and nine of them were common between all three traits. In fact, all SNPs that were identified for eChl_A were also identified for either eChl_B or eChl_T, or for all three traits (Fig. 4a). Specifically, 55 % of significant SNPs were in common between eChl_A and eChl_B, 56 % between eChl_B and eChl_T, and 45 % between eChl_A and eChl_T. Since Chl *a* and Chl *b* are synthesized by the same pathway, can be interconverted by a Chl *a*—Chl *b* cycle, and sum to make up the total chlorophyll content [77], this was anticipated and, to some extent, cross-validates the genome-wide association analysis results for the eChl_A, eChl_B, and eChl_T traits. In total, five loci were identified to be common among these three traits, one each on chromosomes 10, 15, 18, 19 and 20. Of the five loci, the loci on chromosome 15, 19, and 20 were located in the vicinity of known chlorophyll related genes (Fig. 2, Table 3). Surprisingly, no known chlorophyll-related genes were located near the loci on chromosomes 10 and 18. Thus, these loci may identify genes that have not yet been implicated in the modulation of chlorophyll content. While the loci on chromosomes 15, 19, and 20 were also identified based on tChl_T and iChl_T, the loci on chromosomes 10 and 18 were not, and therefore may be of particular relevance to chlorophyll content in fully expanded sun-exposed leaves near the top of the canopy and not, or less so, for leaves that are older and/or at different position in the canopy (Figs. 2 and 3). The known chlorophyll related genes found near the loci on chromosomes 15, 19, and 20 that were identified based on eChl_A, eChl_B, eChl_T, tChl_T, and iChl_T, include genes annotated to encode proteins that have magnesium chelatase activity (Chr 15, 19). Magnesium chelatase catalyzes the insertion of Mg^2+^ into protoporphryin IX, which is the first committed step in chlorophyll biosynthesis (earlier steps are in common with the heme biosynthetic pathway) [78].

The remaining 19 loci that were identified based on extract-based chlorophyll traits were marked by 34 SNPs, and a search for chlorophyll related genes identified 15 genes in their vicinity (±3 MB). Given how closely related the chlorophyll traits are, more confidence and greater importance can be given to loci that were identified based on more than one trait. These included two loci identified based on three chlorophyll traits (eChl_B, eChl_T and eChl_R (Chr 5) and eChl_A, eChl_T and eChl_R (Chr 8)), and 8 loci that were identified based on two chlorophyll traits. The remaining 9 loci were based on single extract-based chlorophyll traits (Fig. 2) (Additional file 3: Table S3).

Among the chlorophyll-related genes found in the vicinity of the putative loci, chlorophyll A-B binding proteins (8 genes near 8 loci) were the most prominent, followed by genes encoding proteins with magnesium chelatase activity (7 genes near 7 loci) (Table 3). However, the search for chlorophyll-related genes did not reveal hits near every putative locus. This includes the aforementioned loci on chromosomes 10 and 18, that were identified by Chl_A, eChl_B, and eChl_T as well as five additional loci on chromosomes 4, 5, 8, 9 and 19 that were marked by one or a combination of other eChl-based traits. Interestingly, the eChl_R-based locus on chromosome 1 and chromosome 6, were located close to two and one leaflet chlorophyll content QTL, respectively, that were previously identified [33] based on a biparental mapping population. One chlorophyll-related gene, recently cloned [79] as “Stay-Green (SGR) gene D2”, controls the stay-green phenotype in soybean and is involved in regulation of chlorophyll degradation. Recently, Campbell et al. [31] cloned a magnesium chelatase subunit located on chromosome 15, near the first of two loci associated with eChl_R, and Reed et al. [32] identified gene involved in the biogenesis of Photosystem I and II near the second eChl_R locus on chromosome 15, which was also close to a chlorophyll content QTL previously identified by Hao et al. [34]. Both of these genes were identified in distinct chlorophyll deficient mutants. Another eChl_R-based-locus on chromosome 15 was found near a QTL identified by Hao et al. [34] and the QTLs for mutant’s y9 and y17 identified by Palmer and Xu [80] that condition green/chlorotic foliage. The eChl_A and eChl_T-based locus on chromosome 7 was also located close to one leaflet chlorophyll content QTL previously identified by Li et al. [33]. In addition, one of the eChl_A and eChl_T-based loci on chromosome 4 as well as the eChl_T-based locus on chromosome 16 were located close to chlorophyll content QTL previously identified [34] using SNP markers.

### Putative loci for eChl_T, tChl_T, and iChl_T and known chlorophyll genes in their vicinity

Total chlorophyll content was mapped based on leaf-level (eChl_T) and canopy-level estimates (tChl_T and iChl_T). In total, 64 SNPs, 52 of which were unique, were identified for total chlorophyll content based on these three phenotypes. These SNPs identify 27 putative loci in 16 chromosomal regions (Additional file 2: Table S2). Among significant SNPs, 22 % were in common between eChl_T and tChl_T, 33 % between tChl_T, and iChl_T, and 30 % between eChl_T, and iChl_T. The R^2^ values for total chlorophyll loci identified in this study were higher (3.7 to 6.1 %) than the R^2^ values (2.0 to 4.9 %) reported by Hao et al. (2012) [34]. A search for chlorophyll-related genes resulted in 33 candidate genes in the vicinity (±3 MB) of these 52 unique candidate SNPs (Table 4). The chromosomal locations of the 52 SNPs and 33 candidate genes are shown in Fig. 3. As for extract based chlorophyll traits, the most common chlorophyll related genes found in the vicinity of the putative loci were genes encoding chlorophyll A-B binding proteins (10 genes near 9 loci) and genes encoding proteins with magnesium chelatase activity (7 genes near 7 loci) (Table 4).

Four putative loci, one each on chromosomes 8, 15, 19 and 20 were common for all three total chlorophyll phenotypes, thus imparting particular confidence in the validity of these loci (Additional file 2: Table S2). As mentioned above, the loci on chromosomes 15, 19, and 20 were also detected based on eChl_A and eChl_B phenotypes. In contrast to the loci on chromosomes 15, 19, and 20, no known chlorophyll related gene was identified in the vicinity of the locus on chromosome 8, despite having been identified by eChl_A, eChl_B, eChl_T, tChl_T and iChl_T phenotypes (Figs. 2 and 3, Fig. 3).

Of the remaining 23 loci for total chlorophyll content, only three were identified by associations using two methods of chlorophyll determination. One of these, on chromosome 19, was found identified using eChl_T and iChl_T as well as eChl_A, and was located in the immediate vicinity of a gene annotated as magnesium chelatase (Table 4). The other two loci were located on chromosomes 10 and 18 and were both identified with the two canopy spectral reflectance-based traits. While no known chlorophyll-related gene was found near the locus on chromosome 18, two genes (Coenzyme F420 hydrogenase and Protoporphyrinogen oxidase) were found near the locus on chromosome 10. Of the remaining 20 loci identified by single canopy reflectance-based traits, 13 had at least one chlorophyll related gene nearby (Fig. 3). Also, the locus identified based on iChl_T on chromosome 2 was near a QTL for a viable yellow mutant identified by Espinosa [81] and near a chlorophyll content QTL identified by Hao et al [34]. A QTL for a yellow leaf (y10) mutant identified [82], was located near the second tChl_T locus identified on chromosome 3. Interestingly, one QTL identified by Li et al. [33] and one identified by Hao et al. [34], were also located near that same putative locus on chromosome 3 that was also located in the vicinity of a magnesium chelatase (Fig. 3). Two loci for iChl_T on chromosome 11 and 14 respectively were found near chlorophyll content QTLs previously identified by Hao et al. [34].

## Conclusions

Significant variation in chlorophyll content (μg cm^-2^) was observed among the 332 MG IV soybean genotypes examined in this study. Genome-wide association analysis identified putative loci associated with each of six chlorophyll traits examined. Twenty-four unique putative loci on 14 chromosomes were identified for extract-based chlorophyll traits. For total chlorophyll content, determined using three methods, association analyses identified 27 putative loci on 16 chromosomes. Several of the loci were identified by more than one chlorophyll trait and since the traits are closely related, more confidence and greater importance can be given to loci that were identified by more than one trait. While many of the putative loci identified were located near genes previously identified or annotated as related to chlorophyll traits, numerous SNPs marked chromosomal regions where no known chlorophyll-related genes were found. Putative chlorophyll-related loci identified based on high-throughput amenable canopy spectral reflectance characteristics indicate that canopy spectral reflectance can provide useful phenotypes for genome-wide association mapping.

## Abbreviations

BLUP, best linear unbiased prediction; DAP, days after planting; eChl_A, extract-based chlorophyll *a*; eChl_B, extract-based chlorophyll *b*; eChl_R, extract-based chlorophyll a/b ratio; eChl_T, extract-based total chlorophyll; FDR, false discovery rate; GWAS, genome-wide association study; iChl_T, total chlorophyll based on spectral reflectance index; MLM, mixed linear model; MLR, multiple linear regression; QTLs, quantitative trait loci; SNPs, single nucleotide polymorphisms; tChl_T, total chlorophyll based on wavelet transformed spectra

## Additional files

Additional file 1: Table S1. List of significant SNP markers for extractable chlorophyll *a* (eChl_A), chlorophyll *b* (eChl_B), total chlorophyll (eChl_T) and chlorophyll *a/b* ratio (eChl_R). (XLSX 17 kb) Additional file 2: Table S2. List of significant SNP markers identified based on three chlorophyll determination methods namely extractable chlorophyll (eChl_T), and wavelet transformed spectral reflectance total chlorophyll (tChl_T) and spectral reflectance index total chlorophyll (iChl_T). (XLSX 16 kb) Additional file 3: Table S3. List of the 43 nearest genes to the 43 significant SNP markers for extractable chlorophyll *a* (eChl_A), chlorophyll *b* (eChl_B), total chlorophyll (eChl_T) and chlorophyll *a/b* ratio (eChl_R). (DOCX 25 kb) Additional file 4: Table S4. List of the 52 nearest genes to the 52 significant SNP markers identified based on three chlorophyll determination methods namely extractable chlorophyll (eChl_T), wavelet transformed spectral reflectance total chlorophyll (tChl_T) and spectral reflectance index total chlorophyll (iChl_T). (DOCX 26 kb)
