# Supplementary material for: Genome-wide association mapping of soybean chlorophyll traits based on canopy spectral reflectance and leaf extracts
Source: BMC Plant Biol. 2016 Aug 4;16:174. doi: 10.1186/s12870-016-0861-x (PMC4973047; doi:10.1186/s12870-016-0861-x)
Supplement: Additional file 3: Table S3. — List of the 43 nearest genes to the 43 significant SNP markers for extractable chlorophyll a (eChl_A), chlorophyll b (eChl_B), total chlorophyll (eChl_T) and chlorophyll a/b ratio (eChl_R). (DOCX 25 kb) [file 12870_2016_861_MOESM3_ESM.docx]

| **Loci** | **SNP ID** | **Williams 82 allele** | **Alternative allele** | **Position in the gene** | **Gene ID** | **Functional Annotation** | **Trait** |
| --- | --- | --- | --- | --- | --- | --- | --- |
| 1 | BARC_1.01_Gm_01_52570528_T_C | T | C |  | Glyma01g40910 | integral to nuclear inner membrane | eChl_R |
|  | BARC_1.01_Gm_01_52792053_G_A | G | A | Intron | Glyma01g41210 | Intracellular Ribosomal L37ae protein family | eChl_R |
| 2 | BARC_1.01_Gm_04_382134_A_G | A | G |  | Glyma04g00810 | No functional annotation available | eChl_A and eChl_T |
| 3 | BARC_1.01_Gm_04_45147931_C_A | C | A |  | Glyma04g38830 | Predicted E3 ubiquitin ligase | eChl_A and eChl_T |
| 4 | BARC_1.01_Gm_04_8798975_T_C | T | C |  | Glyma04g10600 | Phospholipid binding | eChl_R |
|  | BARC_1.01_Gm_04_8810653_A_C | A | C | CDS | Glyma04g10610 | Zinc finger, C3HC4 type (RING finger) | eChl_R |
| 5 | BARC_1.01_Gm_05_1717646_T_C | T | C | Intron | Glyma05g02330 | No functional annotation available | eChl_B and eChl_T |
|  | BARC_1.01_Gm_05_2630283_A_G | A | G |  | Glyma05g03440 | DNA replication factor C complex | eChl_R |
| 6 | BARC_1.01_Gm_05_9081936_A_G | A | G |  | Glyma05g09341 | Microtubule severing protein katanin p80 subunit B (contains WD40 repeats) | eChl_A and eChl_T |
| 7 | BARC_1.01_Gm_06_13539126_C_T | C | T |  | Glyma06g17200 | U5 snRNP-specific protein-like factor and related proteins | eChl_R |
| 8 | BARC_1.01_Gm_07_17362808_A_G | A | G | Intron | Glyma07g17600 | Cytosolic purine 5-nucleotidase-related (Glyma 1.0) | eChl_A and eChl_T |
| 9 | BARC_1.01_Gm_07_35736677_G_A | G | A | CDS | Glyma07g30770 | Serine-threonine protein kinase, plant-type (Glyma 1.0) | eChl_R |
| 10 | BARC_1.01_Gm_08_42324395_T_G | T | G |  | Glyma08g42321 | Protein dimerization activity | eChl_A and eChl_T |
|  | BARC_1.01_Gm_08_43416504_G_A | G | A |  | Glyma08g43630 | Glycerophosphodiester phosphodiesterase activity | eChl_R |
| 11 | BARC_1.01_Gm_09_800177_A_G | A | G |  | Glyma09g01310 | No functional annotation available | eChl_A and eChl_T |
| 12 | BARC_1.01_Gm_10_17750171_G_A | G | A |  | Glyma10g15250 | N-acetyltransferase activity | eChl_B and eChl_T |
| 13 | BARC_1.01_Gm_10_40258740_G_A | G | A | CDS | Glyma10g31790 | Uncharacterized conserved protein TEX2, contains PH domain | eChl_A and eChl_T |
| 14 | BARC_1.01_Gm_10_4416883_T_C | T | C | Intron | Glyma10g05620 | E3 ubiquitin protein ligase activity | eChl_A eChl_B and eChl_T |
| 15 | BARC_1.01_Gm_15_11382431_A_G | A | G |  | Glyma15g14930 | lipid metabolic process | eChl_B and eChl_T |
|  | BARC_1.01_Gm_15_11384008_T_G | T | G |  | Glyma15g14930 | lipid metabolic process | eChl_B |
|  | BARC_1.01_Gm_15_11647696_A_G | A | G |  | Glyma15g15220 | Uncharacterized conserved protein, contains WD40 repeat and BROMO domains | eChl_B |
|  | BARC_1.01_Gm_15_11712082_A_G | A | G |  | Glyma15g15270 | Protein predicted to be involved in carbohydrate metabolism | eChl_A and eChl_B |
| 16 | BARC_1.01_Gm_15_3023905_C_T | C | T |  | Glyma15g04311 | PPR repeat | eChl_R |
| 17 | BARC_1.01_Gm_15_48027033_G_A | G | A |  | Glyma15g41011 | protein dimerization activity | eChl_R |
|  | BARC_1.01_Gm_15_48028533_G_A | G | A |  | Glyma15g41011 | protein dimerization activity | eChl_R |
| 18 | BARC_1.01_Gm_16_27901019_C_A | C | A |  | Glyma16g24110 | Auxin responsive protein | eChl_T |
| 19 | BARC_1.01_Gm_17_12050653_A_G | A | G |  | Glyma17g15350 | oxidation-reduction process (2OG-Fe(II) oxygenase superfamily) | eChl_R |
| 20 | BARC_1.01_Gm_18_9284632_A_G | A | G |  | Glyma18g10410 | Cotton fibre expressed protein | eChl_A eChl_B and eChl_T |
|  | BARC_1.01_Gm_18_9433511_C_T | C | T | CDS | Glyma18g10660 | Dipeptidyl peptidase III-related | eChl_A eChl_B and eChl_T |
|  | BARC_1.01_Gm_18_9474722_G_T | G | T | CDS | Glyma18g10660 | Dipeptidyl peptidase III-related | eChl_A, eChl_B and eChl_T |
|  | BARC_1.01_Gm_18_9483569_A_G | A | G |  | Glyma18g10625 | ATP binding (Apotoptic process) | eChl_A and eChl_T |
|  | BARC_1.01_Gm_18_9603626_A_C | A | C |  | Glyma18g10750 | Zinc finger protein-related | eChl_A eChl_B and eChl_T |
|  | BARC_1.01_Gm_18_9625531_A_G | A | G |  | Glyma18g10750 | Zinc finger protein-related | eChl_A and eChl_T |
|  | BARC_1.01_Gm_18_9676741_G_A | G | A |  | Glyma18g10750 | Zinc finger protein-related | eChl_A eChl_B and eChl_T |

**Additional File 3 Table S3** List of 43 nearest genes to the 43 significant SNP markers for extractable chlorophyll *a* (eChl_A), chlorophyll *b* (eChl_B), total chlorophyll (eChl_T) and chlorophyll *a/b* ratio (eChl_R).

Continued

| **Loci** | **SNP ID** | **Williams 82 allele** | **Alternative allele** | **Position in the gene** | **Gene ID** | **Functional Annotation** | **Trait** |
| --- | --- | --- | --- | --- | --- | --- | --- |
| 21 | BARC_1.01_Gm_19_36789644_A_G | A | G | CDS | Glyma19g29190 | 26S proteasome regulatory complex, subunit PSMD10 | eChl_A eChl_B and eChl_T |
| 22 | BARC_1.01_Gm_19_47069443_T_C | T | C | CDS | Glyma19g40730 | No functional annotation available | eChl_A and eChl_T |
|  | BARC_1.01_Gm_19_47089771_A_C | A | C |  | Glyma19g40770 | oxidation-reduction process | eChl_A and eChl_T |
| 23 | BARC_1.01_Gm_19_5687583_C_T | C | T |  | Glyma19g05260 | Metal ion transport (Heavy-metal-associated domain) | eChl_R |
|  | BARC_1.01_Gm_19_5690696_T_C | T | C |  | Glyma19g05260 | Copper transport protein atox1-related | eChl_R |
| 24 | BARC_1.01_Gm_20_45190334_G_A | G | A |  | Glyma20g37298 | Domain of unknown function (DUF1981) | eChl_A eChl_B and eChl_T |
|  | BARC_1.01_Gm_20_45228564_G_T | G | T |  | Glyma20g37298 | Guanyl-Nucleotide exchange factor | eChl_B and eChl_T |
|  | BARC_1.01_Gm_20_45432499_G_A | G | A | CDS | Glyma20g37571 | DNA repair (Endonuclease activity) | eChl_A and eChl_T |
|  | BARC_1.01_Gm_20_45515007_C_T | C | T |  | Glyma20g37720 | Uncharacterized conserved protein | eChl_A and eChl_T |

^1^Glycine max (Gm) followed by chromosome number, genomic location and alleles

^2^Methods for which SNP was found to be significantly associated (eChl_A, eChl_B, eChl_T and eChl_R)

^3^Name of Gene is based on information in Soybase Glyma 1.1

^4^CDS- coding DNA sequence; NA- Not applicable
