# Supplementary material for: Genome-wide association mapping of soybean chlorophyll traits based on canopy spectral reflectance and leaf extracts
Source: BMC Plant Biol. 2016 Aug 4;16:174. doi: 10.1186/s12870-016-0861-x (PMC4973047; doi:10.1186/s12870-016-0861-x)
Supplement: Additional file 4: Table S4. — List of the 52 nearest genes to the 52 significant SNP markers identified based on three chlorophyll determination methods namely extractable chlorophyll (eChl_T), wavelet transformed spectral reflectance total chlorophyll (tChl_T) and spectral reflectance index total chlorophyll (iChl_T). (DOCX 26 kb) [file 12870_2016_861_MOESM4_ESM.docx]

**Supporting Information File 4 Table S4** List of 52 genes closest to the 52 significant SNP markers identified based on three total chlorophyll determination methods, namely extractable total chlorophyll (eChl_T), and wavelet transformed spectral reflectance total chlorophyll (tChl_T) and spectral index total chlorophyll (iChl_T).

| **Loci** | **SNP ID** | **Williams 82 allele** | | **Alternative allele** | | **Position in the gene** | **Gene ID** | **Functional Annotation** | **Trait** |
| --- | --- | --- | --- | --- | --- | --- | --- | --- | --- |
| 1 | BARC_1.01_Gm_02_47790307_C_T | C | | T | | CDS | Glyma02g42830 | Protein of unknown function (DUF2985) | iChl_T |
|  | BARC_1.01_Gm_02_47798190_T_C | | T | C | |  | Glyma02g42850 | Metal Ion Binding | iChl_T |
|  | BARC_1.01_Gm_02_47802231_T_C | | T | C | | CDS | Glyma02g42860 | No functional annotation available | iChl_T |
| 2 | BARC_1.01_Gm_03_38359850_C_T | | C | T | | CDS | Glyma03g30410 | Nucleoporin-related | tChl_T |
| 3 | BARC_1.01_Gm_03_7856828_A_G | | A | G | |  | Glyma03g07380 | Thiamine pyruvate decarboxylase [EC:4.1.1.1] | tChl_T |
| 4 | BARC_1.01_Gm_04_382134_A_G | | A | G | |  | Glyma04g00810 | No functional annotation available | eChl_T |
| 5 | BARC_1.01_Gm_04_45147931_C_A | | C | A | |  | Glyma04g38830 | Predicted E3 ubiquitin ligase | eChl_T |
| 6 | BARC_1.01_Gm_05_1717646_T_C | | T | C | | Intron | Glyma05g02330 | No functional annotation available | eChl_T |
| 7 | BARC_1.01_Gm_05_41445765_A_G | | A | G | | CDS | Glyma05g37980 | Mitotic checkpoint protein MAD1 | tChl_T |
|  | BARC_1.01_Gm_05_41455888_A_G | | A | G | |  | Glyma05g37980 | Mitotic checkpoint protein MAD1 | tChl_T |
|  | BARC_1.01_Gm_05_41469190_A_G | | A | G | | 3UTR | Glyma05g38000 | Regulatory protein MLP and related LIM proteins | tChl_T |
| 8 | BARC_1.01_Gm_05_9081936_A_G | | A | G | |  | Glyma05g09341 | Microtubule severing protein katanin p80 subunit B (contains WD40 repeats) | eChl_T |
| 9 | BARC_1.01_Gm_06_50142804_G_A | | G | A | |  | Glyma06g47740 | Phosphoenolpyruvate dikinase-related | tChl_T |
| 10 | BARC_1.01_Gm_07_17362808_A_G | | A | G | | Intron | Glyma07g17600 | Cytosolic purine 5-nucleotidase-related (Glyma 1.0) | eChl_T |
| 11 | BARC_1.01_Gm_08_42321866_A_G | | A | G | |  | Glyma08g42321 | DNA binding and protein dimerization activity | tChl_T |
|  | BARC_1.01_Gm_08_42324395_T_G | | T | G | |  | Glyma08g42321 | Protein dimerization activity | eChl_T, tChl_T and iChl_T |
| 12 | BARC_1.01_Gm_08_7135505_G_A | | G | A | |  | Glyma08g09950 | Eukaryotic cytochrome b561 | iChl_T |
| 13 | BARC_1.01_Gm_09_4833261_T_C | | T | C | |  | Glyma09g06085 | No functional annotation available | tChl_T |
| 14 | BARC_1.01_Gm_09_800177_A_G | | A | G | |  | Glyma09g01310 | No functional annotation available | eChl_T |
| 15 | BARC_1.01_Gm_10_34990419_T_C | | T | C | |  | Glyma10g26640 | Intracellular transport (nucleic acid binding) | tChl_T and iChl_T |
| 16 | BARC_1.01_Gm_10_40258740_G_A | | G | A | | CDS | Glyma10g31790 | Uncharacterized conserved protein TEX2, contains PH domain | eChl_T |
| 17 | BARC_1.01_Gm_10_4416883_T_C | | T | C | | Intron | Glyma10g05620 | E3 ubiquitin protein ligase activity | eChl_T |
| 18 | BARC_1.01_Gm_11_9393129_G_A | | G | A | | CDS | Glyma11g13190 | Family of unknown function (DUF716) | iChl_T |
| 19 | BARC_1.01_Gm_14_10298490_C_T | | C | T | |  | Glyma14g11780 | Endosomal membrane proteins, EMP70 | iChl_T |
|  | BARC_1.01_Gm_14_10299101_C_T | | C | T | |  | Glyma14g11730 | DNA-binding protein C1D involved in regulation of double-strand break repair | iChl_T |
|  | BARC_1.01_Gm_14_10301411_T_C | | T | | C |  | Glyma14g11780 | Endosomal membrane proteins, EMP70 | iChl_T |
|  | BARC_1.01_Gm_14_10304752_T_C | | T | | C |  | Glyma14g11780 | Endosomal membrane proteins, EMP70 | iChl_T |
| 20 | BARC_1.01_Gm_15_11382431_A_G | | A | | G |  | Glyma15g14930 | Lipid metabolic process | eChl_T |
|  | BARC_1.01_Gm_15_11712082_A_G | | A | | G |  | Glyma15g15270 | Protein predicted to be involved in carbohydrate metabolism | eChl_T, tChl_T and iChl_T |

Continued

| **Loci** | **SNP ID** | **Williams 82 allele** | **Alternative allele** | **Position in the gene** | **Gene ID** | **Functional Annotation** | **Trait** |
| --- | --- | --- | --- | --- | --- | --- | --- |
| 21 | BARC_1.01_Gm_16_27901019_C_A | C | A |  | Glyma16g24110 | Auxin responsive protein | eChl_T |
| 22 | BARC_1.01_Gm_18_13066700_G_A | G | A |  | Glyma18g13574 | Regulation of transcription, DNA-dependent | tChl_T |
|  | BARC_1.01_Gm_18_13080290_C_T | C | T |  | Glyma18g13586 | Drug transmembrane transporter activity | tChl_T |
|  | BARC_1.01_Gm_18_13087922_T_C | T | C |  | Glyma18g13586 | Drug transmembrane transporter activity | iChl_T |
|  | BARC_1.01_Gm_18_13112227_T_G | T | G |  | Glyma18g13610 | Iron/ascorbate family oxidoreductases | iChl_T |
| 23 | BARC_1.01_Gm_18_9284632_A_G | A | G |  | Glyma18g10410 | Cotton fibre expressed protein | eChl_T |
|  | BARC_1.01_Gm_18_9433511_C_T | C | T | CDS | Glyma18g10660 | Dipeptidyl peptidase III-related | eChl_T |
|  | BARC_1.01_Gm_18_9474722_G_T | G | T | CDS | Glyma18g10660 | Dipeptidyl peptidase III-related | eChl_T |
|  | BARC_1.01_Gm_18_9483569_A_G | A | G |  | Glyma18g10625 | ATP binding (Apotoptic process) | eChl_T |
|  | BARC_1.01_Gm_18_9603626_A_C | A | C |  | Glyma18g10750 | Zinc finger protein-related | eChl_T |
|  | BARC_1.01_Gm_18_9625531_A_G | A | G |  | Glyma18g10750 | Zinc finger protein-related | eChl_T |
|  | BARC_1.01_Gm_18_9676741_G_A | G | A |  | Glyma18g10750 | Zinc finger protein-related | eChl_T |
| 24 | BARC_1.01_Gm_19_36789644_A_G | A | G | CDS | Glyma19g29190 | 26S proteasome regulatory complex, subunit PSMD10 | eChl_T, tChl_T and iChl_T |
| 25 | BARC_1.01_Gm_19_47069443_T_C | T | C | CDS | Glyma19g40730 | No functional annotation available | eChl_T and iChl_T |
|  | BARC_1.01_Gm_19_47089771_A_C | A | C |  | Glyma19g40770 | Oxidation-reduction process | eChl_T |
| 26 | BARC_1.01_Gm_20_34934762_C_T | C | T | Intron | Glyma20g25180 | Heat shock protein binding (DnaJ superfamily) | tChl_T |
| 27 | BARC_1.01_Gm_20_45190334_G_A | G | A |  | Glyma20g37298 | Domain of unknown function (DUF1981) | eChl_T |
|  | BARC_1.01_Gm_20_45228564_G_T | G | T |  | Glyma20g37298 | Guanyl-Nucleotide exchange factor | eChl_T |
|  | BARC_1.01_Gm_20_45432499_G_A | G | A | CDS | Glyma20g37571 | DNA repair (Endonuclease activity) | eChl_T, tChl_T and iChl_T |
|  | BARC_1.01_Gm_20_45460065_T_C | T | C |  | Glyma20g37611 | Integral to membrane (Signal peptidase complex) | tChl_T |
|  | BARC_1.01_Gm_20_45515007_C_T | C | T |  | Glyma20g37720 | Uncharacterized conserved protein | eChl_T, tChl_T and iChl_T |
|  | BARC_1.01_Gm_20_45686932_T_G | T | G | Intron | Glyma20g37940 | Fatty-acyl-CoA binding | tChl_T |
|  | BARC_1.01_Gm_20_45704109_G_A | G | A |  | Glyma20g37970 | ATP-dependent helicase activity | tChl_T |

^1^Glycine max (Gm) followed by chromosome number, genomic location and alleles

^2^Methods for which SNP was found to be significantly associated (eChl_A, eChl_B, eChl_T and eChl_R)

^3^Name of Gene is based on information in Soybase Glyma 1.1

^4^CDS- coding DNA sequence; NA- Not applicable
